# Posterolateral fusion combined with posterior decompression shows superiority in the treatment of severe lumbar spinal stenosis without lumbar disc protrusion or prolapse: a retrospective cohort study

**DOI:** 10.1186/s13018-020-1552-8

**Published:** 2020-01-22

**Authors:** Chenxu Wang, Xiang Yin, Liang Zhang, Xin Xue, Yu Xiang, Huaijian Jin, Mingyong Liu, Jianhua Zhao

**Affiliations:** Department of Spine Surgery, Center for Orthopedics, Daping Hospital, Army Medical University, No. 10, Changjiangzhilu, Daping, Yuzhong District, Chongqing, 400042 China

**Keywords:** Lumbar spinal stenosis, Posterolateral fusion, Circumferential fusion, Outcome, Complication

## Abstract

**Background:**

Currently, discectomy and posterior decompression combined with lumbar circumferential fusion (CF) have been accepted as a major procedure for severe lumbar spinal stenosis (LSS). However, studies on severe LSS without protruded intervertebral disc to minimize study bias are lacking. We aimed to investigate the effectiveness of sole posterior decompression with lumbar posterolateral fusion (PLF) and the necessity of discectomy and CF in patients with severe LSS without lumbar disc protrusion or prolapse.

**Methods:**

This retrospective cohort study included 153 severe LSS patients without lumbar disc protrusion or prolapse who were admitted in a tertiary spine center with at least a 2-year follow-up between January 2014 and August 2017. Patients were divided into the PLF (*n* = 77; those who underwent posterior decompression with PLF in 1–3 segments) or CF (*n* = 76; those who underwent posterior decompression and discectomy with CF in 1–3 segments) groups. Pedicle screw instrumentation was applied to avoid postoperative instability. Clinical outcomes were assessed by visual analog scale (VAS), Oswestry Disability Index (ODI), and Japanese Orthopedic Association Score (JOA, lumbar pain score). Duration of operation, blood loss, surgical cost, and postoperative complications were analyzed. Height of intervertebral space, lumbar lordosis, and bone union were confirmed by lumbar radiography or computed tomography.

**Results:**

Both groups achieved significant improvement in JOA, ODI, and VAS compared with preoperative values (*P* < 0.001), but without significant difference between the two groups. Both groups achieved high fusion rate without difference and correction of lumbar lordosis and intervertebral space height (*P* < 0.001), especially in the CF group (*P* < 0.05). Duration of operation, blood loss, and operation cost were significantly higher in the CF group than in the PLF group (*P* < 0.001). Eight complications were found in both groups (1, PLF group; 7, CF group; *P* < 0.05).

**Conclusions:**

After posterior decompression, PLF successfully achieves bony fusion and symptom relief with lower complication rate, lesser surgical blood loss, shorter operative time, and lesser cost than CF. Thus, sole posterior decompression with PLF is an effective treatment for severe LSS without lumbar disc protrusion or prolapse.

## Background

Lumbar spinal stenosis (LSS) is a degenerative condition in which changes in the disc, ligamentum flavum, and facet joints along with aging cause narrowing of the spinal canal, producing symptoms of pain in the legs and back, as well as impair ambulation and other disabilities, and it is a common finding in an aging or degenerative spine. LSS often begins with changes in the intervertebral disc, leading to intervertebral space height diminishment. Accompanying intervertebral space, height loss, stress, and mobility of the facet joints increase. These pathological processes lead to thickening of the ligamentum flavum, osteophyte formation, and hypertrophy of facet joints [1–3], causing central canal and particularly lateral recess stenosis, resulting in compression of neural elements and a series of syndromes. In partial LSS, although it acts as an initial factor of LSS, the intervertebral disc presents as only degeneration or bulging, which causes mild compression to the dural sac and nerve roots anteriorly, while the posterior compression from the facet joints and ligamentum flavum is dominant [4].

When symptoms become refractory to conservative management, surgical treatment is commonly considered. The primary goal of surgical treatment is to decompress the neural structures that are being encroached upon. However, for LSS with severe lumbar central canal and lateral recess stenosis, minimal invasive surgery and traditional laminotomy are technologically difficult and may not decompress the spinal canal completely. Therefore, open surgical removal of the inferior 2/3 lamina, inferior articular process, and partially hypertrophic and cohesive superior articular process (it is the decompression range of transforaminal lumbar interbody fusion) is necessary [5]. However, more than 75% of unilateral or bilateral facetectomy can cause iatrogenic instability to the lumbar spine [6–9]; thus, supplement with lumbar fusion and pedicel screw fixation is necessary for minimizing the potential risk of future instability and deformity.

Combination of lumbar interbody fusion (LF) and lumbar posterolateral fusion (PLF), commonly referred to as 360° fusion or circumferential fusion (CF), has become popular since the mid-1970s, with the aim to increase the fusion rate for iatrogenic or degenerative lumbar instability [10]. Currently, many investigators have reported the advantages and disadvantages of CF and PLC, but the bias existing in most studies has attenuated the power of reliability. The most important bias lies in the diverse degenerative conditions of study subjects, such as lower back pain, isthmic or degenerative spondylolisthesis, lumbar disc herniation, lumbar instability, lumbar spinal stenosis, and so on. The inconsistency in the pathological nature of the patient of past studies rendered it difficult to reach convincing consensus on better fusion strategy. Thus, we investigated the necessity of CF in patients with severe LSS but without disc protrusion or prolapse to minimize the bias in the study design and to confirm that posterior decompression and PLF without discectomy has similar clinical efficacy and fewer disadvantages compared to posterior decompression and CF with discectomy.

## Methods

### Materials

All procedures were performed in Daping Hospital, Army Medical University. A total of 153 consecutive patients who underwent decompression, pedicle screw fixation, and either PLF or CF from January 2014 to August 2017 and with minimum follow-up of 2 years were retrospectively studied. The patients were divided into the following two groups: PLF group (posterior decompression with PLF and pedicle screw fixation, *n* = 77) and CF group (posterior decompression, discectomy with CF and pedicle screw fixation, *n* = 76). The two groups were normalized by age, sex, surgical region and level, stenosis degree, smoking status, comorbidity, preoperative symptom [visual analog scale (VAS), Japanese Orthopedic Association score (JOA, lumbar pain score), and Oswestry Disability Index (ODI)], preoperative intervertebral space height, and lumbar lordosis (Table 1).

**Table 1:** Patient data

Parameters	PLF group (*n* = 77)	CF group (*n* = 76)	*P*
Male–female ratio	33 / 44	36 / 40	NS
Patient age: years (range)	63.0 ± 8.4 (49–79)	60.6 ± 7.4 (46–76)	NS
Surgery region: unilateral–bilateral ratio			NS
Unilateral	43	51	
Bilateral	34	25	
Surgical level			NS
No. of patients who underwent one level	46	42	
No. of patients who underwent two level	27	33	
No. of patients who underwent three level	4	1	
Stenosis degree			NS
Grade A	0	0	
Grade B	34	30	
Grade C	35	37	
Grade D	8	9	
Lateral recess stenosis degree (*n*)			NS
Grade 0	0	0	
Grade 1	0	0	
Grade 2	38	35	
Grade 3	39	41	
Medical illness			NS
No. of patients with diabetes	9	6	
No. of patients with cardiovascular	16	12	
No. of patients with others	6	7	
No. of patients who smoke	12	15	NS

Chi-square test (or the Fisher’s exact test when there were counts of < 5) was used for categorical variables. Unpaired sample *t* test was used for continuous variables (patient age)

*NS* no significant difference

### Inclusion and exclusion criteria

**Fig. 1:**
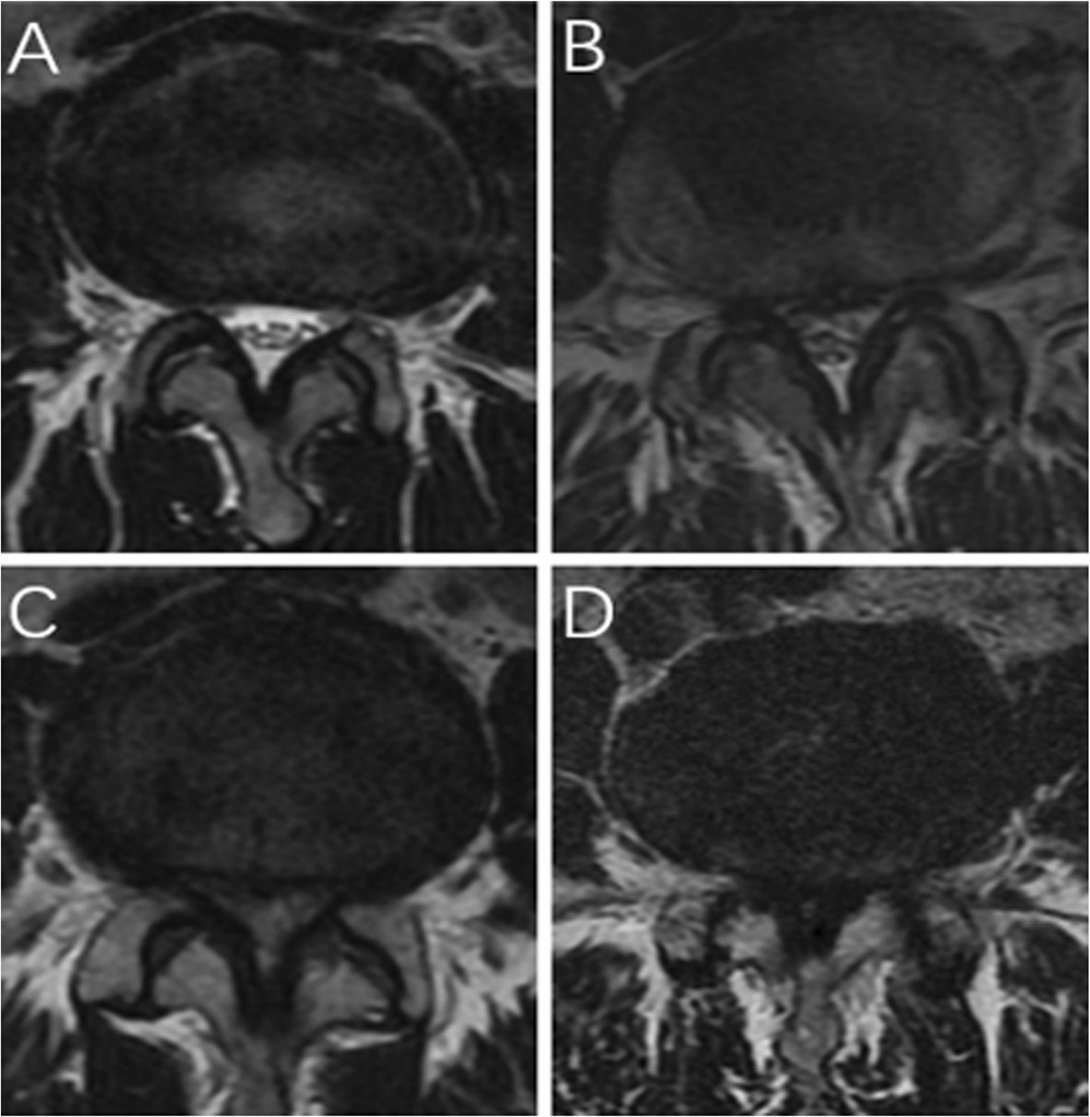
Classification of LSS. **a** Grade A: no or minor stenosis; there is clearly CSF visible inside the dural sac. **b** Grade B: moderate stenosis; the rootlets occupy the whole of the dural sac, but they can still be individualized. Some CSF is still present giving a grainy appearance to the sac. **c** Grade C: severe stenosis; no rootlets can be recognized, the dural sac demonstrating a homogeneous gray signal with no CSF signal visible, and there is epidural fat present posteriorly. **d** Extreme stenosis; in addition to no rootlets being recognizable, there is no epidural fat posteriorly

**Fig. 2:**
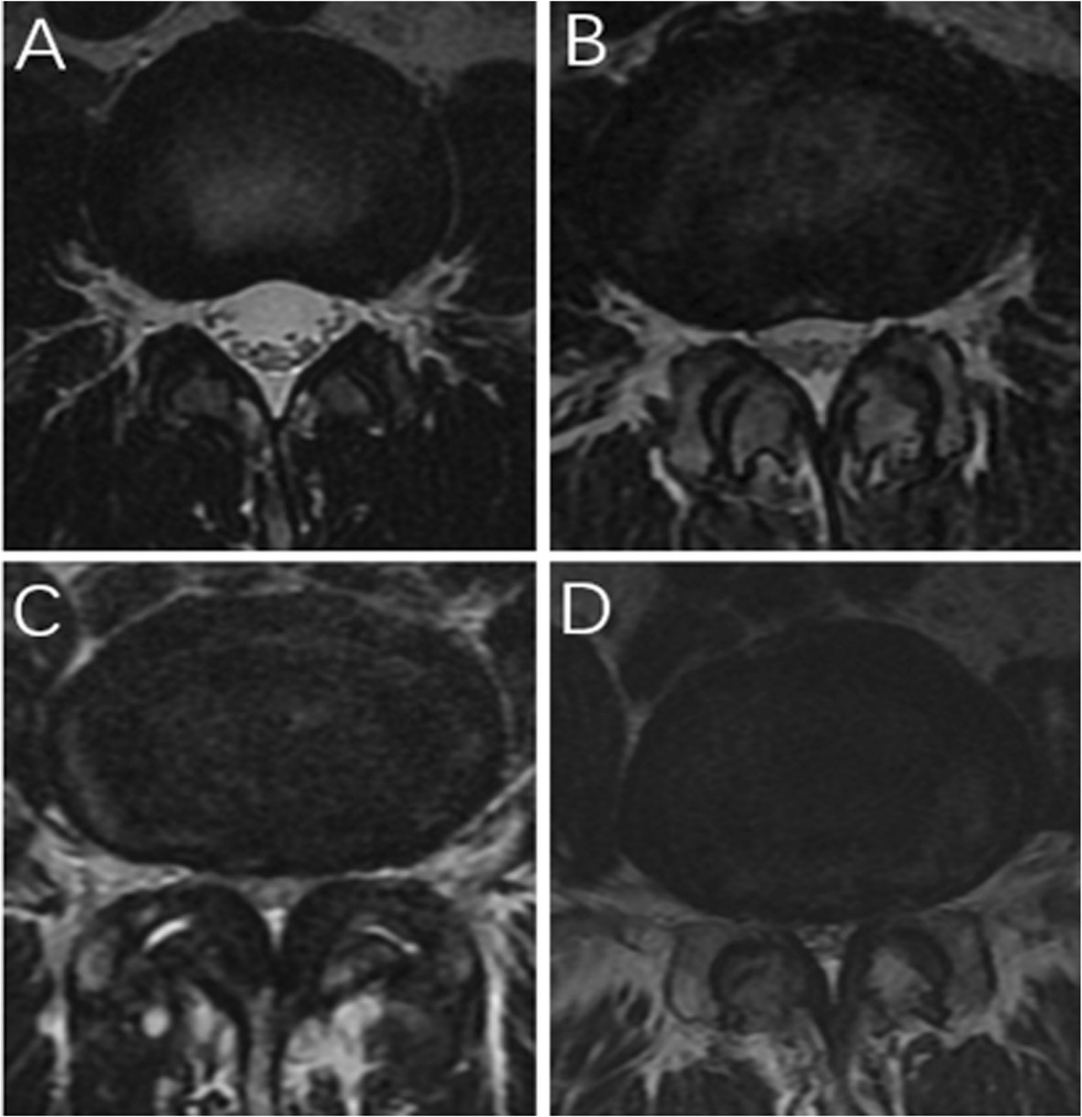
Classification of lateral recess stenosis. **a** Grade 0: normal. **b** Grade 1: the size of lateral recess stenosis is reduced, but the nerve root is not compressed and visualized. **c** Grade 2: the size of lateral recess stenosis is reduced and the nerve root is compressed. **d** Grade 3: severe hypertrophy of the facet and ligamentum flavum, no space or CSF is identified in the lateral recess, and the nerve root is compressed severely

The inclusion criteria were as follows: one to three level degenerative LSS; back and/or leg pain refractory to conservative treatment for at least 6 weeks; LSS of grades B to D based on the morphological characteristic in magnetic resonance imaging (MRI, Fig. 1) [11–13]; and lateral recess stenosis of grades 2–3 in MRI (Fig. 2) [14, 15]. The exclusion criteria included LSS with disc protrusion or prolapse, acute spinal fracture, infection, tumor, revision, spondylolisthesis, and scoliosis (cobb > 20°).

### Surgical techniques

All operations were performed by one experienced spine surgeon, and fusion was done with an autologous bone, which was harvested from the resected laminae and facet joints. Routine exposure and pedicle screw placement were done as previously described [16]. Only the symptomatic side underwent decompression, and the decompression range of the two groups included the unilateral or bilateral inferior 2/3 lamina, inferior articular process, and partially hypertrophic and cohesive superior articular process. In the CF group, the corresponding intervertebral disc was resected (PLF group retained the intervertebral discs). After thorough decompression, a bone graft was placed on the contralateral decorticated laminae and decartilaginified facet joint in the patients who underwent unilateral decompression or on the bilateral decorticated transverse processes in those who underwent bilateral decompression. In the CF group, in addition to the abovementioned steps, the cage (polyetheretherketone) filled with an autologous bone was placed in the intervertebral space for intervertebral fusion. After surgery, all patients wore a rigid plastic corset for 12 weeks.

### Methods

Clinical evaluation was made on the improvement of back pain, leg pain, and disability. Back and leg pain was measured with a 10-point VAS preoperatively, 3 and 6  months postoperatively, and 1 and 2 years postoperatively. Disability was assessed with ODI and JOA (lumbar pain score) preoperatively, 3 and 6 months postoperatively, and 1 and 2 years postoperatively. In addition, duration of operation and surgery blood loss were evaluated, and the complications were also recorded.

Radiologic evaluation included change of the involved height of intervertebral space, lumbar lordosis angle pre- and postoperatively at 6 months and 1 and 2 years. Fusion rate was evaluated postoperatively at 6 months and 1 and 2 years. The height of intervertebral space was measured as follows: a vertical line was made from the midpoint of the upper vertebral inferior endplate to the lower vertebral superior endplate in a lumbar radiography. Lumbar lordosis was evaluated by the angle between the superior edge of the vertebral body of L1 and S1 in a lumbar radiography [17].

**Fig. 3:**
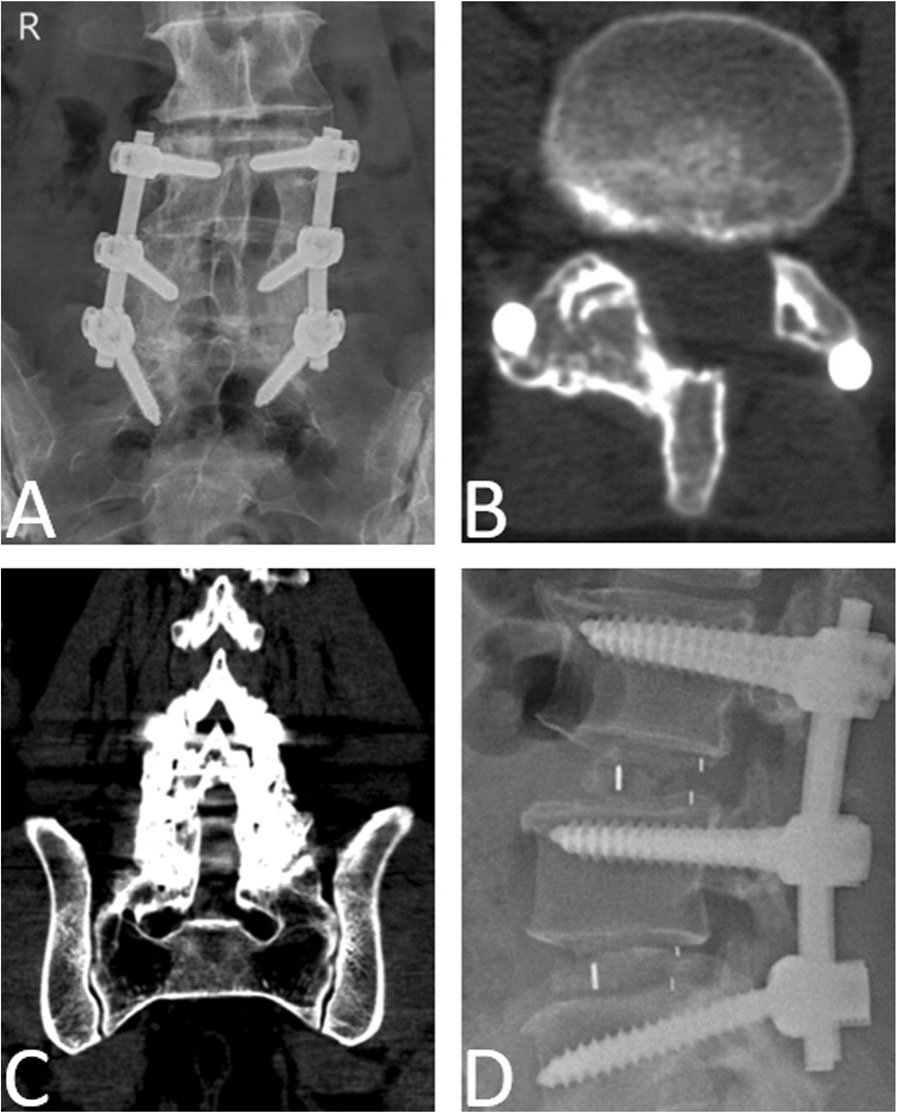
Fusion standard. **a** Lumbar radiography and **b** axial CT showed that a continuous bony bridge had formed between the right L4–S1 laminae and right L4/5, and the L5/S1 facet joint space was replaced by an osseous connection. **c** Anteroposterior CT showed that there were continuous bony bridges between the L4 and S1 transverse processes at the two sides (Grade A). **d** Lumbar radiography showed that there were continuous bony bridges in the L3/4 and L4/5 intravertebral spaces

Fusion was evaluated according to the lumbar radiograph. In the PLF group, for the patients who underwent unilateral decompression, fusion was judged to be present if there was a continuous bony bridge between the contralateral upper and lower laminae, and the facet joint space was replaced by an osseous connection (Fig. 3a, b). For those who underwent bilateral decompression, bone trabecular bridging between the upper and lower transverse processes was scaled according to the degree of bone incorporation, which were as follows: grade A, bilateral; grade B, unilateral; grade C, uncertain; and grade D, resorption [18]. Fusion was judged to be present if there was a continuous bony bridge between the upper and lower transverse processes in at least one side (A or B, Fig. 3c). In CF group, cases conformed to either the posterolateral fusion standard or the intervertebral fusion standard (a solid bar of the bone was present within or anterior to the cages, Fig. 3d) were considered fusion [19]. If the fusion was uncertain, computed tomography was performed to determine the fusion.

All data were entered into the SAS statistical program, version 9.4. For continuous variables, the *t* test was used to calculate statistical significance. Given that the discontinuous variables (VAS, JOA, ODI) were not exactly normally distributed, the Wilcoxon signed rank test was considered appropriate for testing the differences. For categorical variables, chi-square test (or the Fisher’s exact test when there were counts of <  5) was used to examine statistical significance. All results were considered significant if *P* < 0.05.

## Results

### Operation data

Total mean operative time was 133 min (one level: 119 min, two level: 149 min, three level: 180 min) and 162 min (one level: 151 min, two level: 175 min, three level: 200 min) in the PLF and CF groups, respectively. The total average amount of blood loss during the operation was 140 ml (one level: 122 ml, two level: 162 ml, three level: 199 ml) and 192 ml (one level: 169 ml, two level: 218 ml, three level: 250 ml) in the PLF and CF groups, respectively. The operative time and blood loss were significantly lower in the PLF group than in the CF group at any level (*P* < 0.001, Table 2).

**Table 2:** Operation data

Parameters	PLF group (*n* = 77)	CF group (*n* = 76)	*P*
Operative time (min)			
One level (PLF = 46, CF = 42)	118.8 ± 13.0	150.8 ± 13.4	< 0.001
Two level (PLF = 27, CF = 33)	149.3 ± 15.4	174.5 ± 17.6	< 0.001
Three level (PLF = 4, CF = 1)	180.0 ± 8.2	200	< 0.001
Total level	132.7 ± 22.7	161.8 ± 19.7	< 0.001
Blood loss (ml)			
One level (PLF = 46, CF = 42)	122.3 ± 19.9	169.4 ± 24.6	< 0.001
Two level (PLF = 27, CF = 33)	162.0 ± 20.9	217.9 ± 34.1	< 0.001
Three level (PLF = 4, CF = 1)	198.8 ± 31.2	250	< 0.001
Total level	140.2 ± 31.1	191.5 ± 38.1	< 0.001

Values presented as mean ± standard deviation. Unpaired sample *t* test indicated analysis of variance. Differences between the two groups were significant (*P* < 0.001)

**Table 3:** Pain, disability scores, and complications

Parameters	PLF group (*n* = 77)	CF group (*n* = 76)	*P*
Low back pain, VAS score (range)			
Preoperative	6.0 (4.0, 7.0)	5.0 (4.0, 6.0)	NS
3 months	2.0 (1.0, 2.0)	1.0 (1.0, 2.0)	NS
6 months	1.0 (1.0, 1.0)	1.0 (1.0, 1.0)	NS
1 year	1.0 (1.0, 1.0)	1.0 (0, 1.0)	NS
2 years	1.0 (0, 1.0)	1.0 (0, 1.0)	NS
Radicular pain, VAS score (range)			
Preoperative	8.0 (7.0, 8.0)	8.0 (7.0, 8.0)	NS
3 months	1.0 (1.0, 2.0)	1.0 (1.0, 1.0)	NS
6 months	1.0 (1.0, 1.0)	1.0 (0, 1.0)	NS
1 year	1.0 (0, 1.0)	0 (0, 1.0)	NS
2 years	0 (0, 1.0)	0 (0, 1.0)	NS
JOA (range)			
Preoperative	13.0 (13.0, 14.0)	14.0 (13.0, 15.0)	NS
3 months	24.0 (23.0, 24.0)	24.0 (23.0, 25.0)	NS
6 months	25.0 (24.0, 26.0)	25.0 (25.0, 26.0)	NS
1 year	25.0 (24.0, 26.0)	26.0 (25.0, 26.0)	NS
2 years	26.0 (25.0, 26.0)	25.0 (24.0, 27.0)	NS
ODI (range)			
Preoperative	54.0 (52.2, 59.4)	54.9 (50.4, 57.6)	NS
3 months	14.4 (12.6, 16.2)	12.6 (9.0, 16.2)	NS
6 months	10.8 (9.0, 14.4)	12.6 (9.0, 14.4)	NS
1 year	10.8 (7.2, 12.6)	9.9 (7.2, 10.8)	NS
2 years	9.0 (7.2, 10.8)	7.2 (5.4, 10.8)	NS
Postoperative complications (*n*)	1	7	※

Discontinuous variables (VAS, JOA, and ODI) were not exactly normally distributed. Wilcoxon signed rank test was considered appropriate for testing the differences between the two groups and within each group. Fisher’s exact test was used to calculate differences in categorical variables (postoperative complications). VAS, JOA, and ODI were expressed as the median (interquartile range)

*NS* no significant difference

※, *P* < 0.05

### Clinical results

Compared with the preoperative values, back and leg pain of both groups decreased significantly at all measurement timepoints postoperatively (*P* < 0.001), but no statistical difference was found between the two groups (*P* > 0.05). Function parameters, ODI and JOA, both improved significantly in the longitudinal analysis in both groups (*P* < 0.001). However, the differences between the two groups were not statistically significant (*P* > 0.05, Table 3).

### Radiologic results

**Fig. 4:**
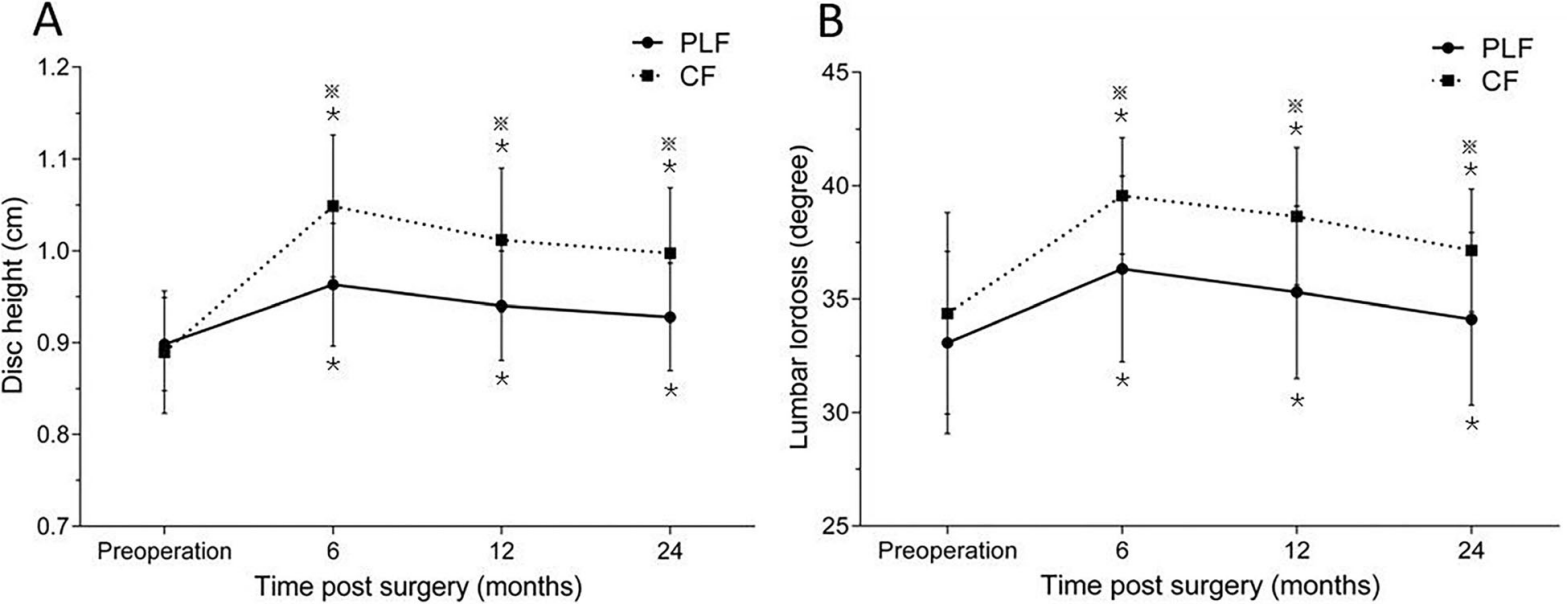
Radiologic data. **a** Disc height and **b** lumbar lordosis in both groups increased significantly compared with the preoperative value, especially in the CF group. ^*^There was a statistical difference compared with the pre-operative value. **※**There was a statistical difference found between the two groups

**Fig. 5:**
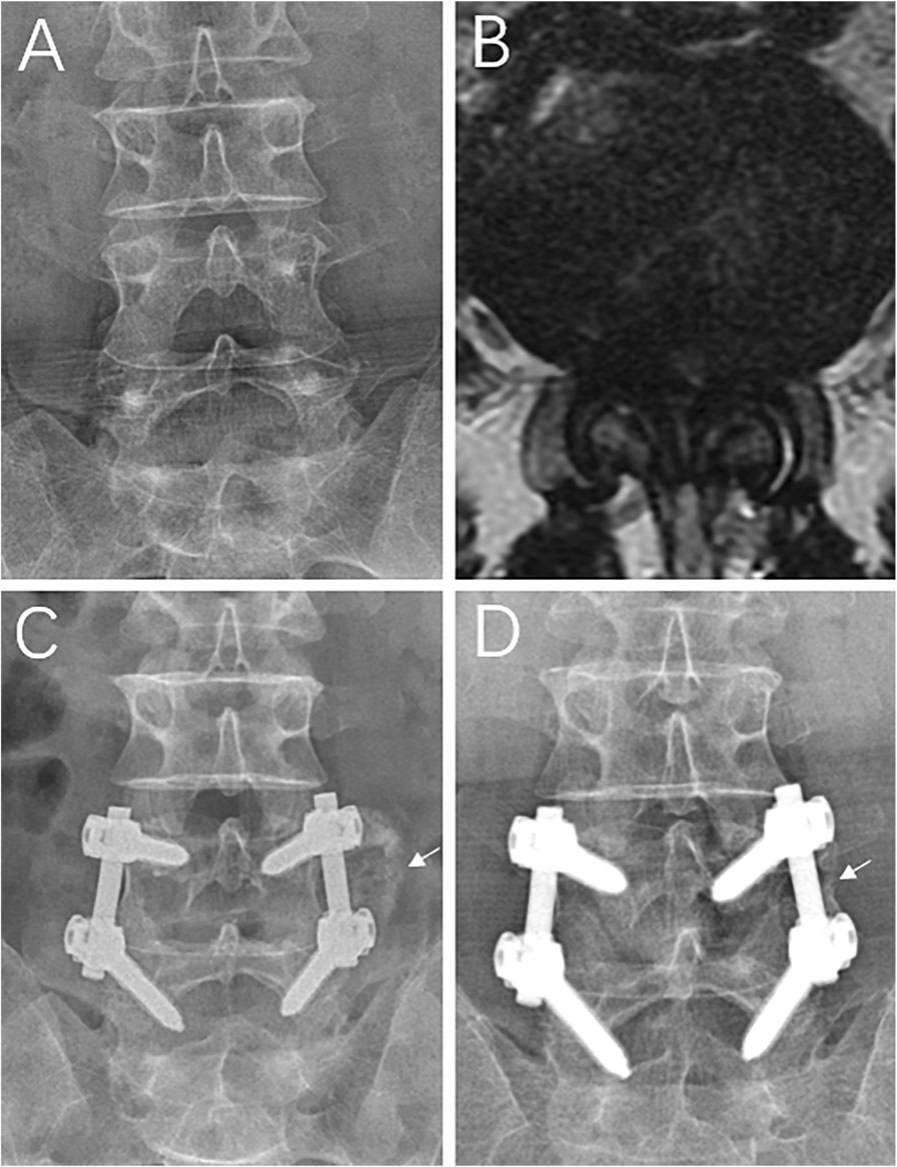
A typical case. A 61-year-old man suffered from L4/5 bilateral spinal canal decompression, fusion, and pedicle screw instrumentation fixation. Before operation, VAS (low back pain) was 5; VAS (radicular pain), 8; JOA, 14; and ODI, 57.6. Two years postoperatively, VAS (low back pain) was 1; VAS score (radicular pain), 1; JOA, 26; and ODI, 10.8. **a** Preoperative lumbar radiography. **b** Preoperative lumbar MRI indicating severe stenosis of the L4/5 spinal canal. **c** Three months after operation, lumbar radiography indicating that the bone graft between the right L4/5 transverse process has been absorbed and that between the left L4/5 transverse process is ossifying. **d** Two years after operation, lumbar radiography indicating that continuous bony bridge has formed between the left L4/5 transverse process

As for the intervertebral space height and lumbar lordosis, both groups showed statistically significant increase at all measurement timepoints compared to their pre-operative values (*P* < 0.001), but the CF group showed an obvious increase in these parameters (*P* < 0.05, Fig. 4). The fusion rate was 80% postoperatively at 6 months, 86% at the first postoperative year, and 92% at the second postoperative year in the PLF group. The corresponding figures were 84%, 92%, and 95% in the CF group, showing no significant difference at all measurement timepoints postoperatively between the two groups (*P* > 0.05). The nonunion rate at the last follow-up was 8% (6 patients) and 5% (4 patients) in the PLF and CF groups, respectively (Table 4). A typical case in the PLF group is shown in Fig. 5.

**Table 4:** Radiologic results

Parameters	PLF group (*n* = 77)	CF group (*n* = 76)	*P*
Disc height (cm)			
Preoperative	0.90 ± 0.05	0.89 ± 0.06	NS
6 months	0.96 ± 0.07	1.04 ± 0.07	※
1 year	0.94 ± 0.06	1.00 ± 0.07	※
2 years	0.93 ± 0.06	0.99 ± 0.07	※
Lumbar lordosis (degree)			
Preoperative	33.08 ± 4.02	34.37 ± 4.44	NS
6 months	36.32 ± 4.09	39.55 ± 2.57	※
1 year	35.30 ± 3.81	38.64 ± 3.04	※
2 years	34.12 ± 3.80	37.14 ± 2.71	※
No fusion (*n*)			
6 months	17	12	NS
1 year	11	6	NS
2 years	6	4	NS

There were no significant differences between the two groups, as calculated with unpaired sample *t* test and Chi-square test

*NS* no significant difference

※, *P* < 0.05

### Complications

**Fig. 6:**
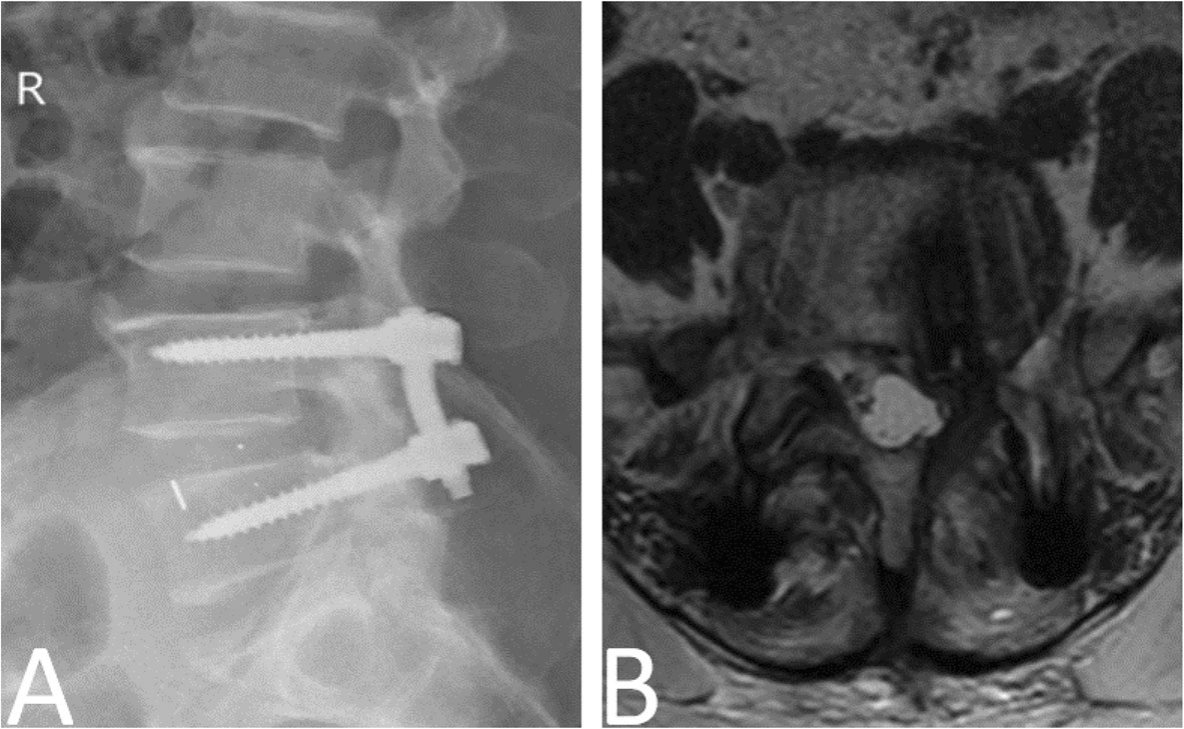
Representative morbidity of the CF group. **a** A lumbar lateral radiograph in the CF group showed that cage of the L4/5 intervertebral space sank into the L5 vertebral body and the L4/5 intervertebral space height was reduced. **b** A postoperative 6-month axial magnetic resonance image in the CF group showed that the cage of the L5/S1 intervertebral space was displaced posteriorly to the lumbar canal and compressed the left S1 nerve root

Postoperative complications in the PLF group included one deep surgical site infection. The CF group had two nerve root injury, two deep surgical site infection, one cerebrospinal fluid leakage, one cage migration, and one cage subsidence case. Thus, the postoperative complication rate was higher in the CF group than in the PLF group (*P* < 0.05). All three patients with infection were treated successfully with debridement and antibiotics. The patient with cerebrospinal fluid leakage was placed in the Trendelenburg position for 10 days to prevent excessive cerebrospinal fluid loss and recovered without incision infection and nerve injury. The two patients with nerve root injury improved spontaneously during the follow-up, and pain was relieved completely at 6 months postoperative. The patient with cage subsidence underwent conservative treatment, but mild low back pain (VAS: 2) remained at the last follow-up (Fig. 6a). However, one patient in the CF group who had L3 to S1 decompression, fixation, and fusion underwent revision surgery because of recurrent pain (VAS: 4) and numbness in the left lower extremity at 16 days postoperatively, and the symptoms were not relieved even after 6 months of conservative treatment. Moreover, MRI showed that the cage was displaced posteriorly to the lumbar canal, which compressed the left S1 nerve root (Fig. 6b). Moreover, the surgery cost was approximately $10,000 and $8500 in the CF and PLF groups, respectively. The lower cost in the PLF group was mainly attributed by the saved cage and shortened surgical and anesthesia duration.

## Discussion

Our findings revealed that after posterior decompression, PLF successfully achieves bony fusion and symptom relief with lower complication rate, lesser surgical blood loss, shorter operative time, and lesser cost than CF, thus indicating that sole posterior decompression with PLF is an effective treatment for severe LSS without lumbar disc protrusion or prolapse.

As a standard procedure, fusion has been conducted for decades to treat instability caused by pathological or iatrogenic causes. The representative two types consist of CF and PLF. Compared to CF, PLF is limited to the posterior parts of the spine, does not involve the anterior column, which simplifies the surgical procedure, and is less technically demanding and avoids extensive dissection, nerve traction, and cage implantation, thus abating surgical blood loss and operative time and preventing a series of postoperative complications.

Recently, for LSS with severe stenosis of the lumbar central canal and lateral recess, discectomy, posterior decompression, and pedicle screw instrumentation fixation combined with CF have been the main surgery methods. Moreover, the two chief reasons to determine postoperative symptoms are complete decompression and postoperative successful fusion, and some investigators believe that discectomy, posterior decompression, and CF are more likely to achieve the above two requirements than PLF [20]. However, in this study, both groups achieved satisfactory back and lower limb pain relief, without no statistical difference, which confirms that sole posterior decompression and PLF is an effective strategy for severe LSS without intervertebral disc protrusion. We believe that the reasons are as follows: first, according to the pathological and anatomical characteristics of LSS, intervertebral disc of most LSS cases only presents as degeneration and bulging, which leads to mild compression to the nerve roots and dural sac anteriorly, so posterior decompression alone is enough to remove the compression; second, even though there is intervertebral disc bulging, after facetectomy and laminectomy, the nerve roots and dural sac can move posteriorly in the lumbar canal to avoid compression; third, PLF can also achieve solid bone fusion compared to CF in the treatment of severe LSS.

In addition, some scholars advocate discectomy because disc degeneration is one of the causes of low back pain, so CF has been considered more advantageous in reducing low back pain than PLF [21, 22]. Moreover, without discectomy, degenerative disc may worsen or protrude in future, which may result in the recurrence of low back pain or compression of the nerve root. However, other investigators have described that discectomy did not increase the relief of low back pain [23]. In this study, the results of the two groups in low back pain and disability were not statistically different, and this outcome was attributable to pedicle screw instrumentation fixation and solid bone fusion between the fusion vertebrae. Solid bone fusion can significantly decrease relative activity between the fusion vertebrae, which reduces stress stimulation toward the corresponding intervertebral disc. Therefore, low back pain can ameliorate significantly and degenerative disc may not aggravate in future [24, 25].

Although with similar outcomes, the CF procedure causes significantly increased operative time and blood loss owing to the additional discectomy and cage implantation, which requires the maneuver of the abundant epidural venous plexus. The CF procedure also results in an increased complication rate. Eight patients (5%) in both groups had postoperative complications, and the CF group showed a higher complication rate than the PLF group. In the CF group, two patients experienced a nerve root injury, which was characterized by leg pain and numbness, that was mainly caused by intraoperative traction of the nerve root to insert the cage, and it was relieved at 6 months postoperatively.

Other serious complications in this study, including one case of cage subsidence and one case of cage retrodisplacement, resulted from the inappropriate placement of the cage. Subsidence may lead to low back pain, mechanical failure of the anterior column support, and loss of sagittal imbalance and disc height [26]. As for cage retrodisplacement, in addition to the abovementioned harms, the neural elements may be compressed if the cage was displaced posteriorly to the spinal canal. We considered that the excessively decartilaginificated endplate and low bone mineral density were the main reasons for subsidence [27, 28]. The relative small size of the cage, osteoporosis, inadequate endplate preparation intraoperatively, and absence of compressing adjacent levels after cage insertion can all result in cage retrodisplacement [29]. In addition, we infer that long segment fixation (two or more cage implantation), which leads to stress decentralization, is also a primary reason, although further study is in needed to confirm this hypothesis.

Currently, some scholars consider that the anterior column is the main loading region; hence, CF is superior to PLF in terms of fusion rate, and CF has an advantage of achieving recovery of lumbar lordosis and intervertebral space height [20, 30]. However, other scholars noted that both CF and PLF are expected to achieve bony fusion and distinct relief of pain [31]. In our study, no significant statistical difference in the fusion rate was found between the two groups and there was no pseudarthrosis. We considered that this finding was due to the the following reasons: first, pedicle screw instrumentations were performed in all cases which supplied a stable environment for bone fusion; second, LSS mostly occurred in the elderly, who had less motion in the spine, than in young people, thereby causing less load to fusion segments; third, PLF was enough to realize the solid bone fusion: as for unilateral decompression patients, the articular space of the facet joint was generally 2–4 mm, so the narrow articular space made the grafting bones easy to fuse. As for bilateral decompression patients, because the intertransversarii was adequate, it had sufficient blood supply, which provided a good environment for the growth of the grafting bone.

## Limitations

There are some limitations in our study. First, this study lacked long-term follow-up. Second, if LSS is combined with intervertebral disc protrusion or prolapse, discectomy combined with CF should be performed for complete decompression and spinal stability. Therefore, appropriate patient selection is needed for each operation.

## Conclusions

In this study, both CF and PLF groups achieved pain relief and bony fusion, but the PLF group showed advantages over CF in terms of shorter operative time, lesser amount of blood loss, fewer complications, and lesser surgical cost. Thus, sole PLF with posterior decompression was considered a preferable method for the treatment of severe LSS without disc protrusion or prolapse.

## Data Availability

The data and materials might be obtained from the corresponding author upon request.

## Abbreviations

CF: Lumbar circumferential fusion
JOA: Japanese Orthopedic Association Score
LF: Lumbar interbody fusion
LSS: Lumbar spinal stenosis
ODI: Oswestry Disability Index
PLF: Lumbar posterolateral fusion
VAS: Visual analog scale
